# Persistent Lower Limb Deformities Despite Amelioration of Rickets in X-Linked Hypophosphatemia (XLH) - A Prospective Observational Study

**DOI:** 10.3389/fendo.2022.866170

**Published:** 2022-03-24

**Authors:** Gabriel T. Mindler, Alexandra Stauffer, Andreas Kranzl, Stefan Penzkofer, Rudolf Ganger, Christof Radler, Gabriele Haeusler, Adalbert Raimann

**Affiliations:** ^1^ Department of Pediatric Orthopaedics, Orthopaedic Hospital Speising, Vienna, Austria; ^2^ Vienna Bone and Growth Center, Vienna, Austria; ^3^ Laboratory for Gait and Movement Analysis, Orthopaedic Hospital Speising, Vienna, Austria; ^4^ MRI Institute Bader, Orthopaedic Hospital Speising, Vienna, Austria; ^5^ Department of Pediatrics and Adolescent Medicine, Division of Pediatric Pulmonology, Allergology and Endocrinology, Medical University of Vienna, Vienna, Austria

**Keywords:** X-linked hypophosphatemia (XLH), deformities, Burosumab, skeletal dysplasia, torsion, phosphate, FGF23.

## Abstract

**Background:**

Gait deviations, lower limb pain and joint stiffness represent key symptoms in patients with X-linked hypophosphatemia (XLH, OMIM 307800), a rare disorder of mineral homeostasis. While the pathomechanism for rickets is well understood, the direct role of PHEX (Phosphate-regulating neutral endopeptidase) deficiency in non-rachitic features including complex deformities, skull and dental affections remains unclear. FGF23-inhibiting antibody treatment can normalize serum phosphate levels and to improve rickets in XLH patients. However, linear growth remains impaired and effects on lower limb deformity and gait are insufficiently studied.

**Aims:**

To characterize and evaluate the course of lower limb deformity in a case series of pediatric XLH patients receiving Burosumab therapy.

**Methods:**

Comparative assessment of planar radiographs, gait analysis, biochemical and clinical features of pediatric patients before and ≥12 months after initiation of FGF23-inhibiting was performed prospectively. Lower limb maltorsion was quantified by torsional MRI and gait analysis. Standardized deformity analysis of lower limb anteroposterior radiographs was conducted.

**Results:**

Seven patients (age 9.0 +/-3.6 years) were eligible for this study. All patients received conventional treatment before onset of antibody treatment. Maltorsion of the femur was observed in 8/14 legs using torsional MRI (mean antetorsion 8.79°). Maltorsion of the tibia was observed in 9/14 legs (mean external torsion 2.8°). Gait analysis confirmed MRI findings with femoral external malrotation prior to and one year after onset of Burosumab therapy. Internal foot progression (intoeing gait) remained pathological in all cases (mean 2.2°). Knee rotation was pathologically internal 10/14 legs. Mean mechanical axis deviation (MAD) of 16.1mm prior to Burosumab changed in average by 3.9mm. Three children underwent guided growth procedures within the observation period. Mild postprocedural rebound of frontal axis deviation was observed under Burosumab treatment in one patient.

**Conclusions:**

This is the first study to investigate lower limb deformity parameters quantitatively in children with XLH receiving Burosumab. One year of Burosumab therapy was associated with persistent maltorsion and frontal axis deviation (varus/valgus) despite improved rickets in this small, prospective uncontrolled study.

## Introduction

X-linked hypophosphatemia (XLH, OMIM #307800) is a rare genetic disorder of renal phosphate wasting leading to profound, chronic hypophosphatemia and skeletal sequelae (1). Phosphate loss and associated rachitic growth plate alterations have been identified as the major cause of lower limb deformity. Accordingly, amelioration of phosphate availability has been regarded as the main therapeutic principle. With availability of Fibroblast-like Growth Factor 23 (FGF23) inhibiting therapies, normalization of phosphate levels can be achieved in most patients with profound improvement of rickets (2).

Lower limb deformity and gait alterations are amongst the main complaints of patients with XLH (3), have been described in affected children and adults (4–6) and are associated with impairment of quality of life (7).

Burosumab has been shown to significantly reduce rachitic changes of the physis, improve serum parameters of bone metabolism and to a lesser extent growth (8, 9). Furthermore, a positive influence on lower limb deformity in children using a lower limb score was described (8–10). However, in these studies, deformity was neither defined in detail nor characterized in a standardized manner, leaving the open question as to whether Burosumab treatment has an impact on standardized parameters of lower limb deformity in children with XLH.

In this study we report preliminary results of lower limb development in children with XLH from our prospective pediatric XLH cohort after at least 1 year of Burosumab treatment.

## Material and Methods

Patients seen in the Vienne Bone and Growth Center receiving Burosumab therapy with a minimum 1-year follow-up were considered for prospective analysis. Follow up included annual anteroposterior long leg standing radiographs (ankle to hip) of both legs, as well as annual instrumented gait analysis and one-time torsional MRI.

10 children received Burosumab treatment at the time of examination. Two patients were excluded due to therapy duration of less than one year and one patient (age 3 years) was excluded due to lack of radiographs and gait analysis data. Follow up radiographic examinations were performed for extended time ranges in patients with guided growth procedures.

Burosumab treatment was initialized and regularly monitored by the pediatric osteologist team. Deformity and gait deviation surveillance was obtained by local pediatric orthopedic protocols. Gait analysis was conducted annually and/or prior to surgical interventions starting at the age of around 4-5 years until skeletal maturity to monitor deformity development non-invasively. A Vicon motion capture system (Vicon, Oxford, United Kingdom) was used during gait analysis, as well as both a modified Cleveland model for movement of the lower extremity and a Plug in Gait model for movement of the upper extremity as marker sets (11, 12). Patient data from a minimum of five force plate strikes per foot were collected during a 12-meter walkway at a self-selected speed. A historic age-matched gait lab group was used as a pediatric control group. Radiographic assessment included a full length anteroposterior (ap) radiograph (hip to ankle) of both lower limbs in standing position with a calibration ball within one month prior to onset of Burosumab and at the 12 months follow up. Deformity analysis consisted of standard measurements of the mechanical axis deviation (MAD), neck shaft angle (NSA) lateral proximal femoral angle (PFA), lateral distal femoral angle (LDFA), medial proximal tibial angle (MPTA) and lateral distal tibial angle (LDTA) (13) and was analyzed using the TraumaCad software (Brainlab AG, Munich, Germany). Rickets severity score (RSS) (14) was obtained by averaged values of two independent, blinded specialists. Comparison between pre-treatment and treatment values was performed by paired Student’s t-test. Normal distribution was tested by Shapiro-Wilk test. A p value of ≤ 0.05 was considered statistically significant.

Evaluation of malalignment using torsional MRI was conducted by calculation of femoral and tibial torsion angles. Torsion of the femur was analyzed by calculating an angle measured between a central femoral head to femoral neck isthmus line and a posterior surface of the femoral condyles (15). For tibial torsion on the other hand, the angle between the posterior condylar axis at the proximal tibia and the bimalleolar axis at the distal tibia was used (16). All measurements were performed by a specialized radiologist (SP) and compared to age matched normal radiographic values [femur (17): tibia (18)] available in literature. The study was approved by a local ethics committee (EK022020).

## Results

In the study period January 2019 until September 2021 a total of 10 children received Burosumab therapy. 7 children had a minimum follow up of 12 months and therefore were included in this study. Extended radiographic observation periods (>12 months follow up) have been included for patients with guided growth procedures (n=3) with an average monitoring period of 19.9 months (range 15.2 - 28).

**Figure 1:**
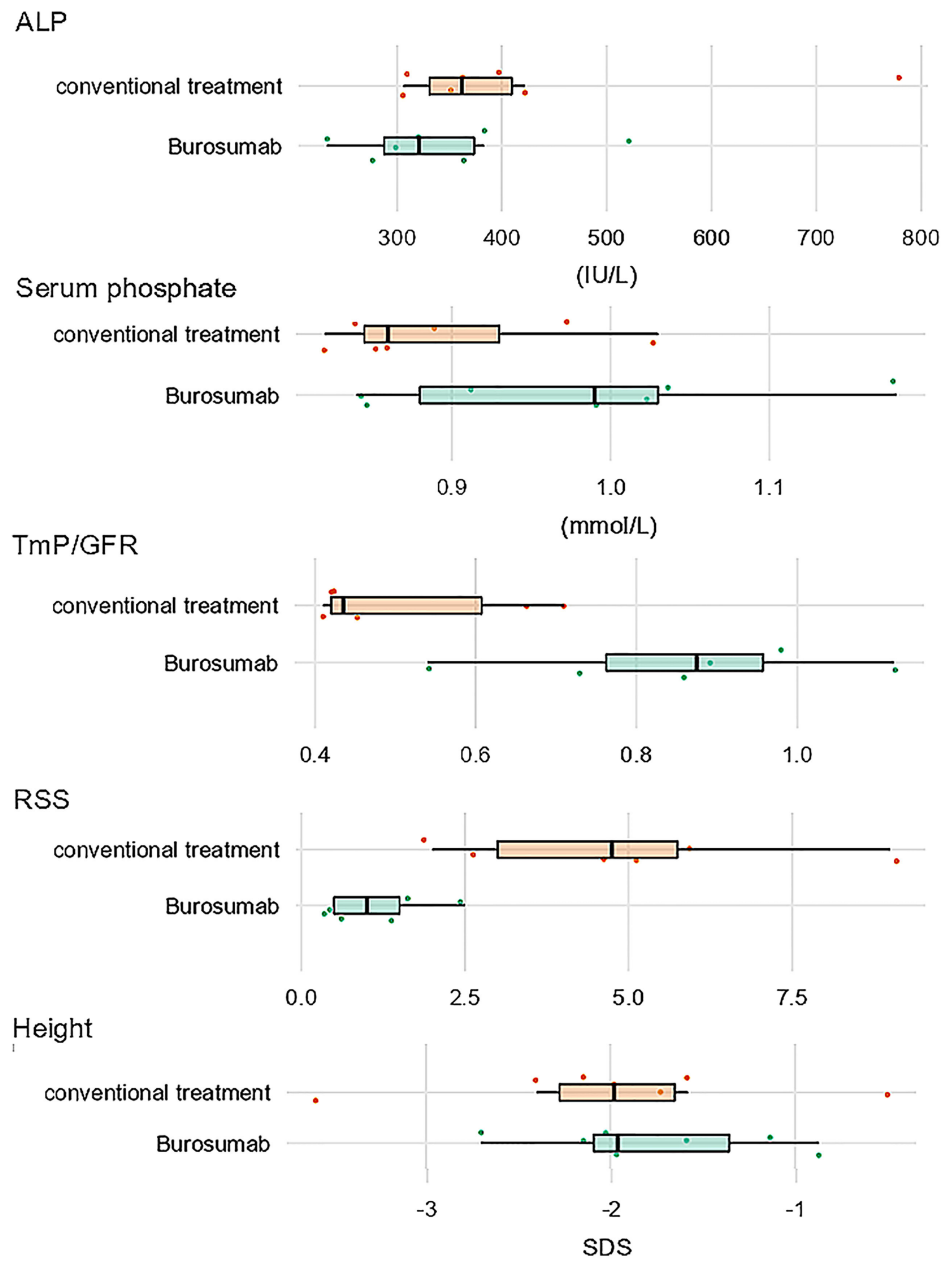
Box plot chart of biochemical, radiographic, and anthropometric markers of treatment response in XLH under conventional treatment (orange) and one year after switch to Burosumab treatment (green). Boxes show median values, upper and lower quartiles.

All children (7 children, 14 legs) showed biochemical improvements under treatment. Radiographic improvements of rachitic changes were observed in 10 of 14 legs with reduced rickets severity scores after 1 year of Burosumab treatment (**Figure 1** and **Supplementary Table 1**). An average increase in body height SDS was observed (0.31+/-0.3 SDS).

### Gait Analysis

**Figure 2:**
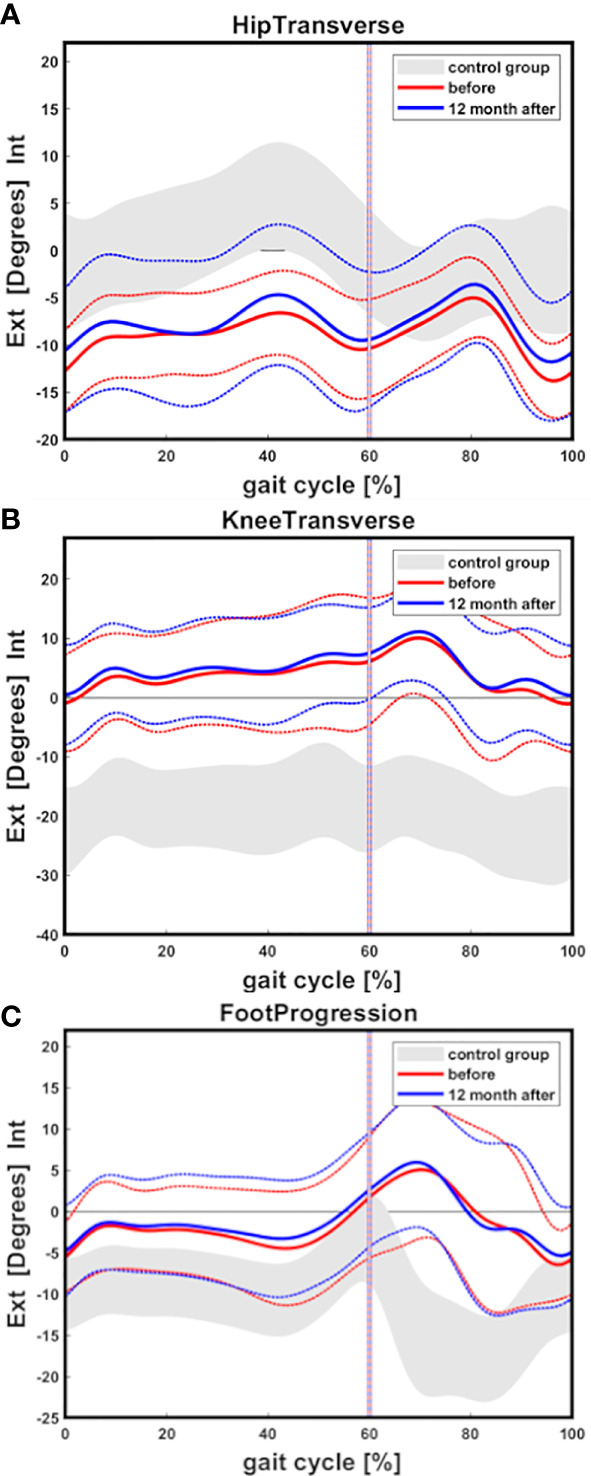
Gait cycle analysis showing overall external hip rotation of children with XLH (n=14 legs) prior to Burosumab (red line) and 12 months after start of Burosumab (blue line) compared to a pediatric control group (gray area) **(A)**. Internal knee rotation **(B)** and internal foot progression **(C)** was observed. Mean value and one standard deviation (dashed line) are presented. Control group band is presented as +/-one standard deviation.

**Figure 3:**
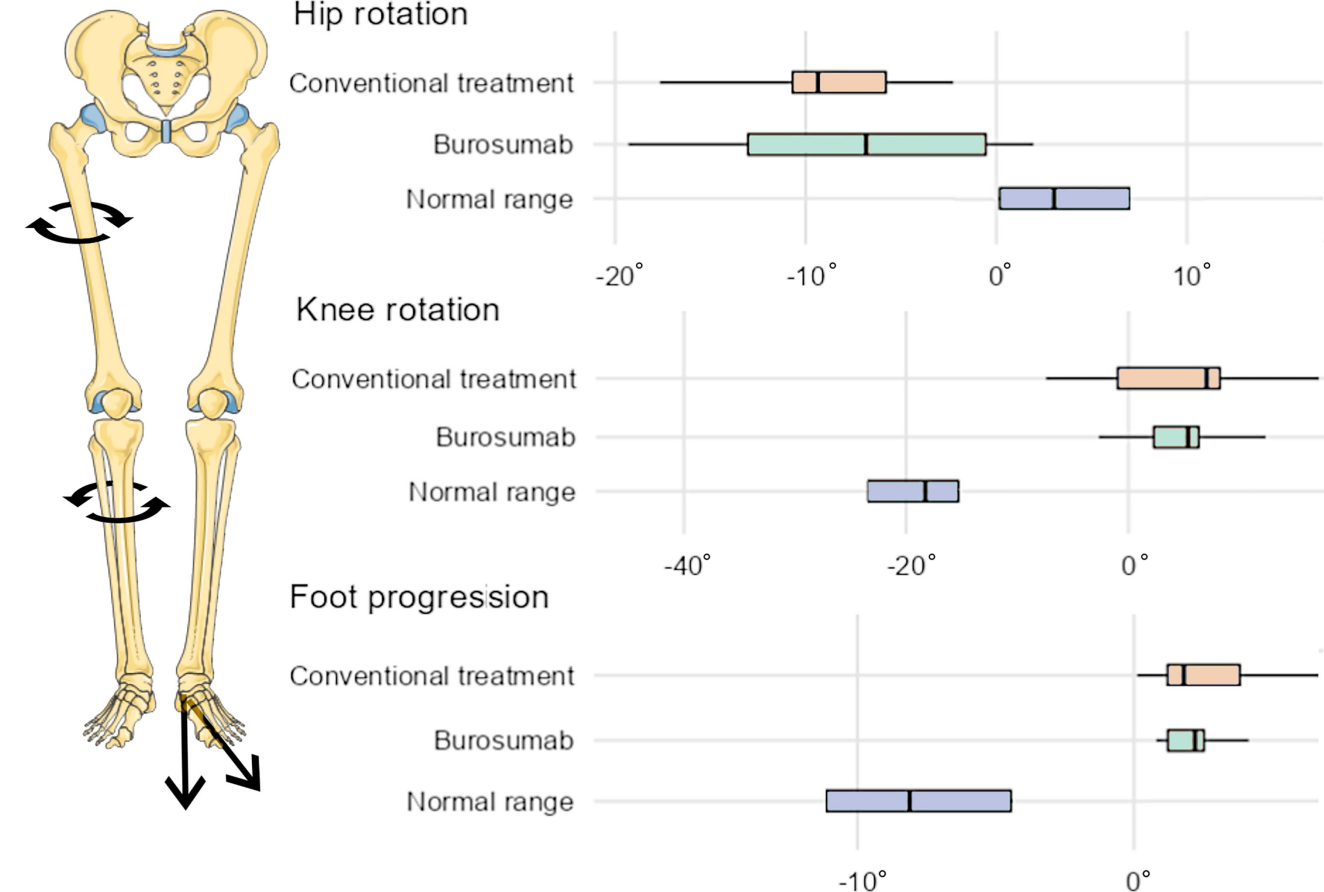
Box chart comparison of gait analysis data (mean rotation during stance) under conventional treatment (orange) and one year after switch to Burosumab treatment (green). X-axis indicates degrees, boxes show median values, upper and lower quartiles. Negative values represent external rotation, positive values internal rotation. Blue boxes represent reference ranges of healthy pediatric controls (Adapted from Servier Medical Art by Servier under a Creative Commons Attribution 3.0 Unported License).

Gait analysis showed external femoral malrotation prior to onset of Burosumab therapy, as well as at the follow-up in all cases (n=14 legs, **Figures 2A**, **3**). The knee rotation was pathologically internal in all but four legs, with no relevant improvement at the follow-up examination (**Figures 2B**, **3**). Internal foot progression (intoeing) with a mean of 2.7° (range 0.1-7.0) remained pathological in all cases [mean 2.2° (0.8-4.1)] independent of age (**Figures 2C**, **3**).

### Radiographic Assessment

Radiographic assessment showed a mean mechanical axis deviation (MAD) of 16.1 mm (range 2-46mm, n=14 legs) prior to onset of Burosumab administration. A change of the MAD of 3.9 mm on average (range 0-12mm) in five children without guided growth during the 12 month follow up period was observed. However, this observation included one case of 12 mm lateral progression. If excluded, the MAD changed merely by 3 mm within a year. Two children were excluded from MAD analysis due to hemiepiphysiodesis within the observation period of one year. In those cases, a correction of deformity of 0.9 degrees on average for both tibia (MPTA) and femur (LDFA) were observed (correction time: 0.8 degrees/month in case 3 and respectively 1.0 degree in case 4; **Table 1**). Two children (3 legs; cases 1 and 5) showed a varus deformity (medial MAD >15mm) with no improvement during Burosumab therapy. Valgus deformity (lateral MAD >10mm) was observed in five legs (cases 3, 4 and 6) requiring cases 3 and 4 to undergo surgical intervention for valgus correction at the time of initiation of Burosumab treatment. Normalization of the mechanical axis was observed at the one-year follow-up in all surgical cases. Lateralization of the mechanical axis of 14 mm occurred in one leg (case 2) despite Burosumab therapy of 10 months, resulting in a valgus deformity, thus necessitating surgical intervention (temporary hemiepiphysiodesis of the proximal medial tibia) (**Table 1**).

**Table 1:** Guided growth correction rates.

	XLH2*	XLH 3	XLH 4	XLH 6	Kim et al.	Sung et al.
Pharmacological treatment	Burosumab	Burosumab	Burosumab	Conventional		
Age at Index Surgery	10y11mo	7y5mo	8y3mo	5y3mo	∅11,8 years	<14 boys, <12 girls
Deformity	Valgus unilateral	Valgus Bilateral	Valgus Bilateral	Varus bilateral	Varus and Valgus	Valgus
No of physis treated	1	4	4	4	154	175
Mean LDFA correction/month		0.8	1.0	0.6		0.71
Mean MPTA correction/month	0.6*	0.8	1.0	0.6		0.40
Correction rate per year	7.2*	9.6	12.0	7.0	6.5	4,8-8,5
Mean MAD correction per month	2.6*	2.9	6.2	2.2		
Total Time of Guided Growth	*	15.0	5.0	20.0		
	* Guided growth procedure ongoing					

Case 6 received conventional therapy at the time of guided growth. Mean correction of degrees per month as well as mean MAD correction in mm per month are listed above. Correction rate per year shows the number of degrees within a year. Reference values for correction rates in children without XLH are shown on the right-hand side (19, 20). Children with XLH with Burosumab treatment show fast correction rate compared to a significantly younger conventional treated child and compared to norm groups in literature. Cases 2, 3 and 4 underwent temporary hemiepiphysiodesis during Burosumab therapy.

ø = average.

### Torsional MRI

**Figure 4:**
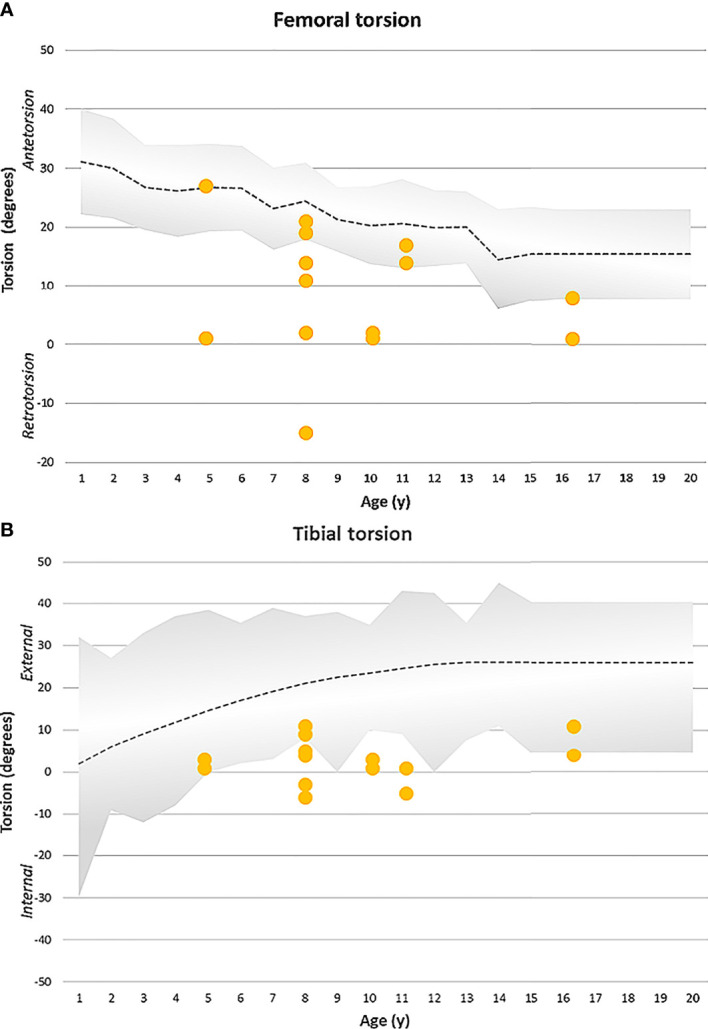
**(A)** Femoral torsion (MRI measurement) observed in the XLH cohort (yellow dots represent each femur) with Burosumab therapy compared to the norm group (grey). 8/14 femora showed reduced antetorsion, respectively retrotorsion. Radiographic norm group values (+-1SD) were obtained from Fabry et al. (17). **(B)** Tibial torsion (MRI) was assessed in a XLH population undergoing Burosumab therapy (yellow dots represent each tibia). 9/14 legs showed reduced external tibial torsion. Clinical reference values (+-2SD) obtained by Staheli et al. (18) are shown by the grey area.

Maltorsion (reduced femoral antetorsion) of femur (mean antetorsion of 8.79°, ranging from 15° retro- to 27° antetorsion, n=14 legs) was observed in 8 of 14 legs using torsional MRI (**Figure 4A**). Maltorsion (decreased external torsion) of the tibia (mean external tibial torsion of 2.8°, range 11° external to 6° internal, n=14 legs) was observed in 9 of 14 legs (**Figure 4B**).

### Case Studies

#### Case 1: 4 Years 2 Months Old Boy, Sporadic Case, Confirmed PHEX Mutation

Lower limb deformity (severe maltorsion of both tibiae) caused frequent falling and the inability to participate in sports (cycling) due to intoeing was reported. Conventional treatment with oral phosphate and alfacalcidol had been initiated at 2.7 years of age. At 4.2 years, treatment was switched to Burosumab (starting dose 0.4mg/kg, 1 year follow-up: 1.7mg/kg) due to persistent rachitic lesions under highest tolerable dosages. Biochemical and radiologic signs of rickets improved drastically under treatment. No relevant frontal plane deformity (varus/valgus) of the lower limbs was found. Linear growth remained stable at -1.9SDS since the beginning of Burosumab treatment. However, no improvement of preexisting maltorsion was detected. Maltorsion was analyzed with gait analysis prior to administration of Burosumab and at the one-year follow-up. Due to persistent gait deviation, preoperative torsion MRI was conducted confirming maltorsion of both tibiae. At the age of 5 years and 5 months the patient underwent surgery with a derotation osteotomy with plate fixation of both tibiae. Full weight bearing was allowed after 4 weeks of cast immobilization and fast healing (after 6-8 weeks) of the osteotomies was achieved.

#### Case 2: 10 Years 1 Month Old Girl, Sporadic Case, Confirmed PHEX Mutation

**Figure 5:**
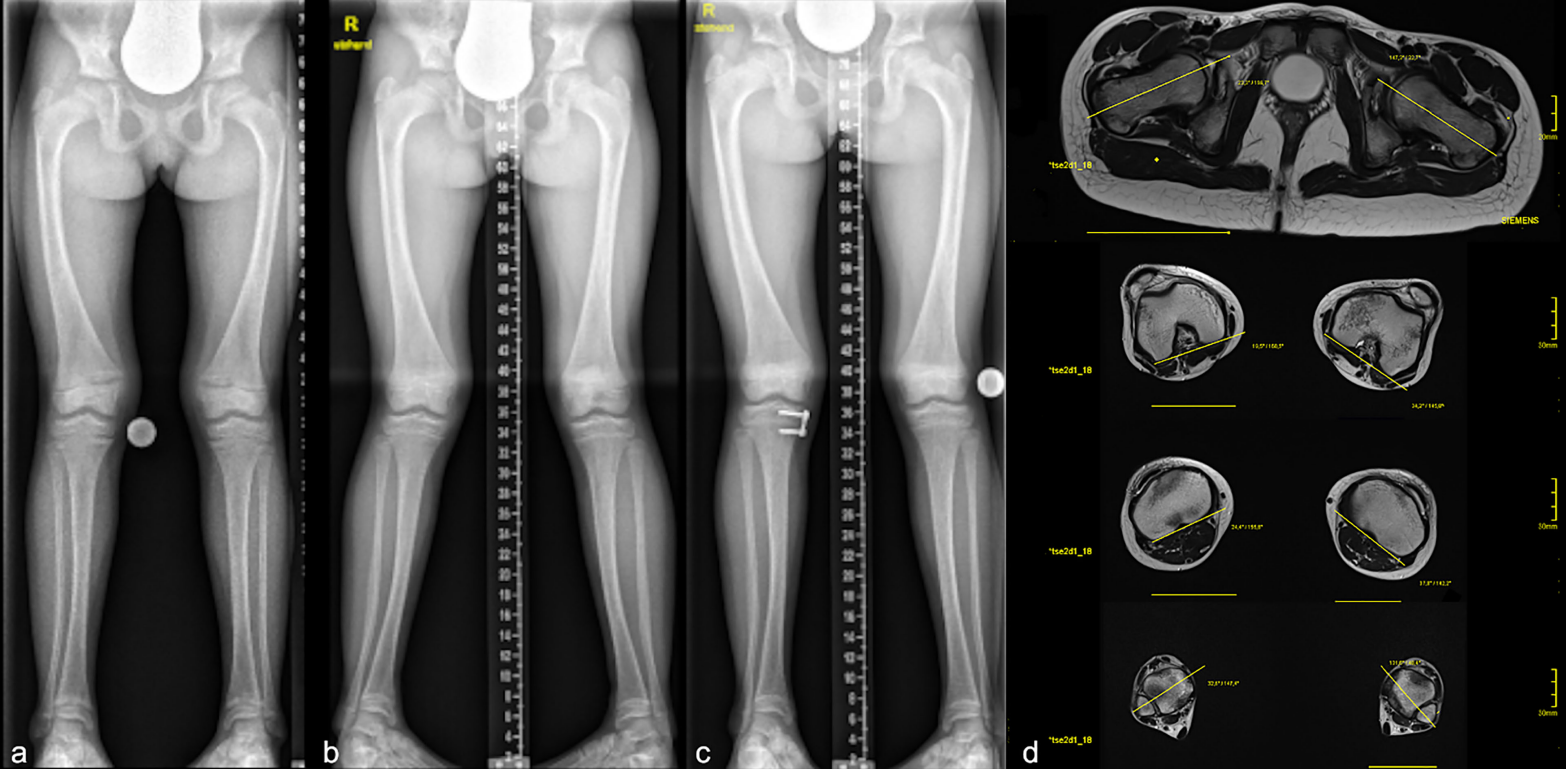
Case 2: Radiographs **(A-C)** and torsional MRI **(D)** of a 10-year-old girl undergoing Burosumab therapy. Full long leg standing radiographs were obtained prior **(A)** to Burosumab initiation and during treatment. Lateralization of MAD in one leg was observed at the 10-month follow-up **(B)**. Guided growth procedure using temporary hemiepiphysiodesis was performed on the right tibia with short term success 6 months postoperative (plate not yet removed) **(C)**. Torsional MRI showed 2° internal torsion of the femur and 3° external torsion of the tibia **(D)**.

**Figure 6:**
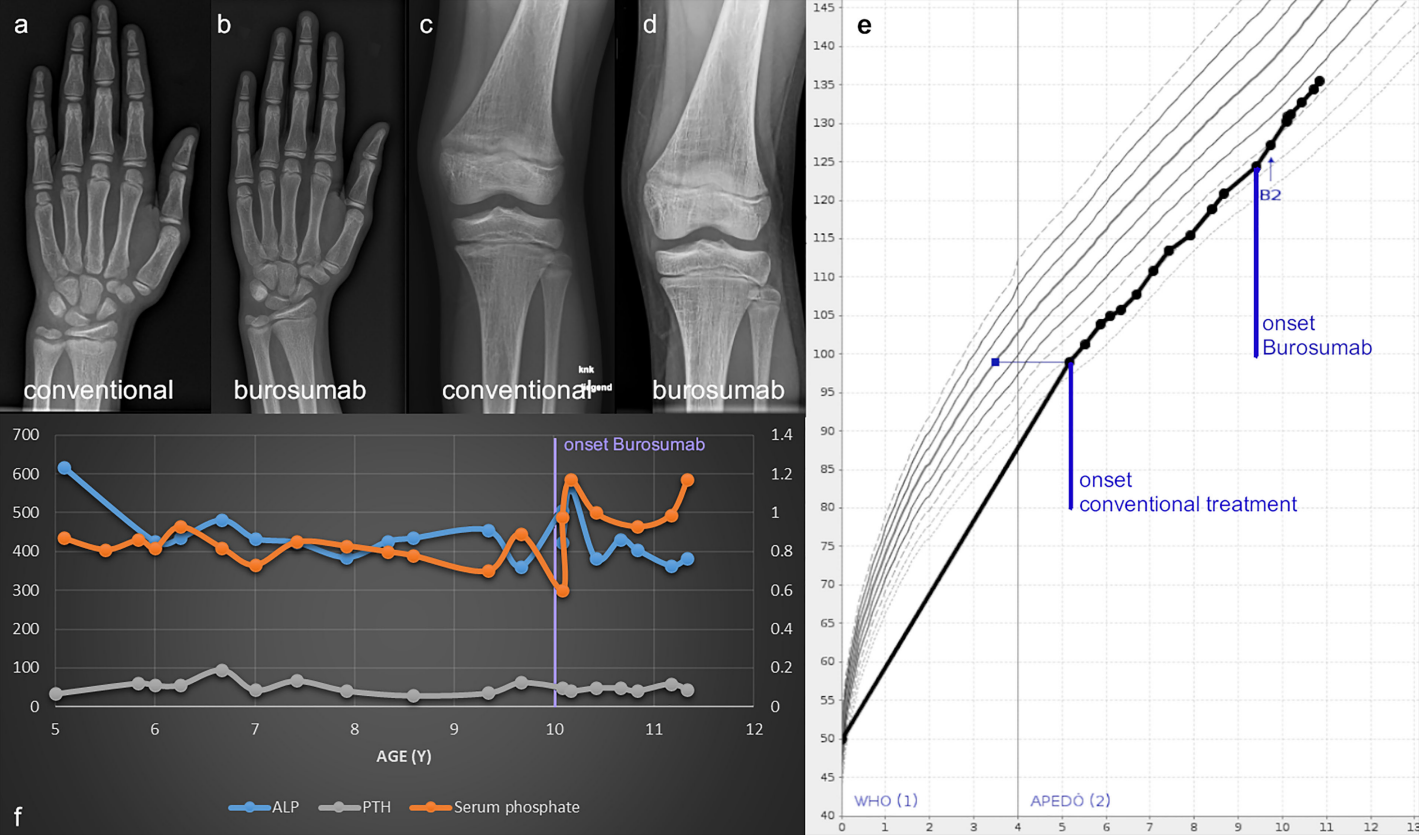
Wrist and knee radiographs obtained before therapy onset **(A, C)** and at the one-year follow-up show resolution of rickets **(B, D)** in case 2. Linear growth, onset of treatment and thelarche (B2) are marked on the Austrian reference growth chart for girls **(E)**. The course of relevant biochemical parameters shows increase of serum Phosphate and normalization of ALP with onset of Burosumab treatment around 10 years of age **(F)**.

Frontal plane deformity development of the right leg despite Burosumab therapy (**Figure 5**) Conventional medical treatment was initiated at 5.2 years of age to be switched to Burosumab at 10 years of age (starting dose 0.4mg/kg, 1 year follow-up: 1.6mg/kg). Regular radiographic follow-ups for frontal lower limb alignment assessment showed a new onset of unilateral genu valgum (lateral mechanical axis deviation of 14 mm) after 10 months of Burosumab treatment. No improvement of tibial maltorsion on clinical examination or gait analysis during Burosumab therapy was observed. Torsion MRI at the 1-year follow-up confirmed the maltorsion observed in the gait analysis. Nevertheless, both laboratory and radiographic analysis of physis showed improvement of rickets (**Figure 6**). The patient underwent guided growth surgery to correct the unilateral genu valgum (temporary hemiepiphysiodesis of the proximal medial tibia) at the time of last follow up with a correction rate of the proximal tibia of 0.6 degrees per month (**Figure 5** and **Table 1**). A lumbothoracic scoliosis (Cobb angle Th4-Th11 30 degrees, Cobb angle Th11-L4 36 degrees) was diagnosed within the study period with ongoing brace therapy.

#### Case 3: 7 Years 2 Months Old Girl, Sporadic Case, Confirmed PHEX Mutation

**Figure 7:**
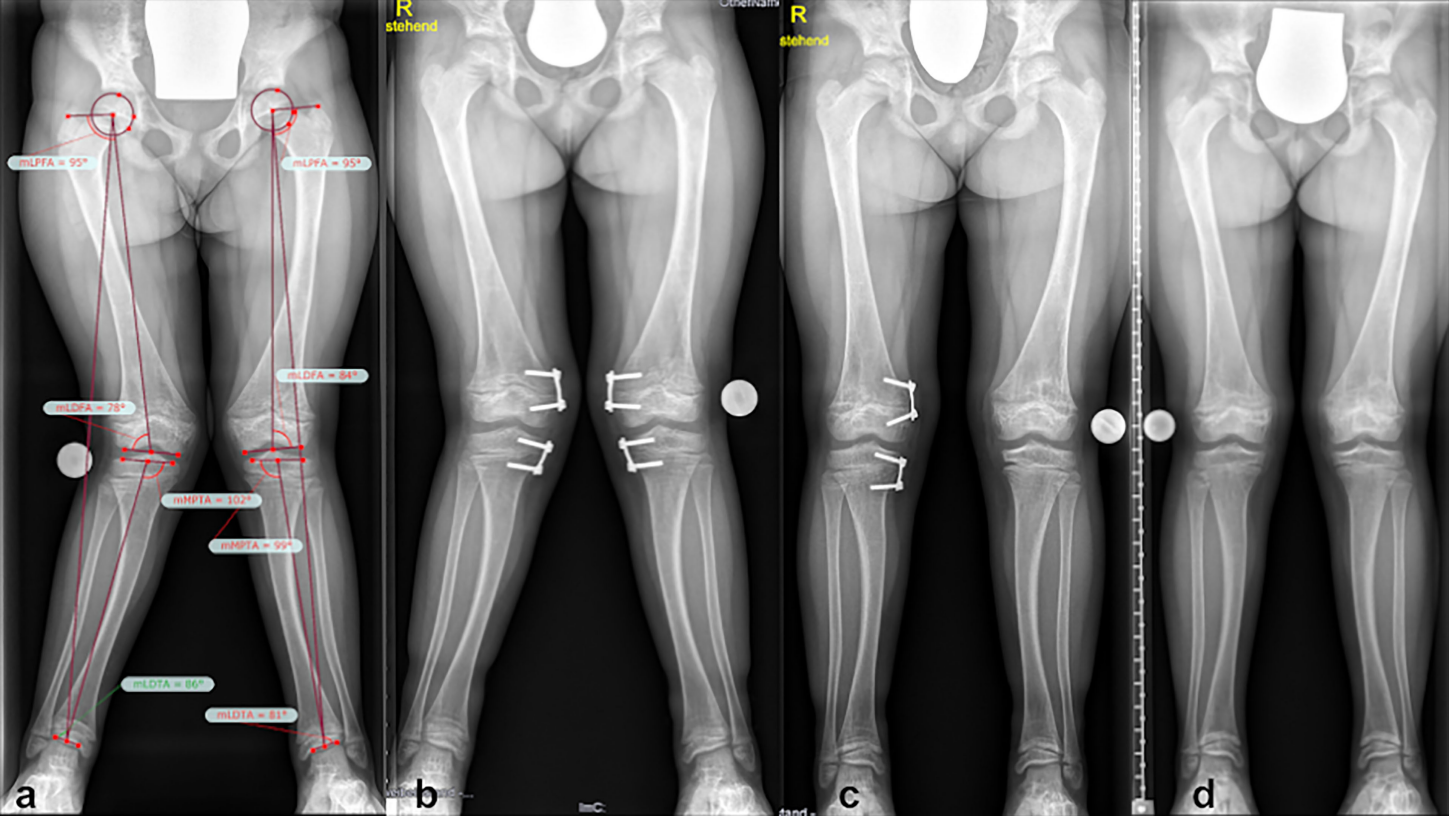
Lower limb deformity in a 7-year-old girl (case 3) undergoing Burosumab therapy. Full long leg standing radiographs and lower limb deformity analysis were obtained prior **(A)** to initiation, as well as during follow-up appointments. Valgus deformity was observed and a temporary hemiepiphysiodesis procedure performed in both legs (x-ray 2 months postoperative) despite persistent rachitic status **(B)**. Normalization of MAD in one leg occurred sooner **(C)**. As a sign of valgus deformity rebound, a mild recurrence of lateralization of the MAD after successful guided growth surgery despite Burosumab therapy was observed at the last follow-up 12 months after plate removal **(D)**.

Mild recurrence of lateralization of mechanical axis (as sign of valgus deformity rebound, rebound of 13mm MAD) after successful guided growth surgery on both legs was noticed on short term postoperative follow up (11 months after removal of guided growth plates) despite Burosumab therapy (starting dose 0.4mg/kg, 1 year follow-up: 1.0mg/kg) (**Figure 7**). Although both laboratory analysis and radiographic evaluation of the physis showed improvement of rickets, no improvement of maltorsion in clinical examination or gait analysis was observed during the 1 year follow up. Guided growth correction rate (Burosumab) was 0.9 degrees per month in the right femur and 0.8 degrees per month in the right tibia, respectively, the left femur corrected 0.7 degrees, whereas the left tibia 0.9 degrees. MAD correction rate was 2.7 mm (right leg) and 3.2 mm (left leg) per month (**Table 1**).

#### Case 4: 7 Years 5 Months Old Girl, Familial Case, First Degree Relative with Confirmed PHEX Mutation

Conventional treatment was commenced during infancy. Therapy was switched to Burosumab at 7.5 years of age due to persistent signs of rickets and onset of hyperparathyroidism during oral phosphate treatment (starting dose 0.8mg/kg, 1 year follow-up: 1.5mg/kg). Frontal plane deformity correction with guided growth was performed at the time of initiation of Burosumab treatment. Fast correction of knee valgus deformity using guided growth (distal medial femur, proximal medial tibia, correction period of only 5 months) with Burosumab treatment was noted. Guided growth correction rate was 1 degree per month on the distal femora, and 1.2 degrees and 0.8 degrees for right and left tibia respectively. MAD correction was 6 mm (right leg) and 6.4 mm (left leg) per month (**Table 1**). No rebound in short term follow-up of guided growth was noticed. While body height and RSS improved (+ 0.5SDS; RSS -X) during 12 months of treatment, maltorsion in clinical examination and gait analysis (MRI verified maltorsion) remained unaltered.

#### Case 5: 15 Years 10 Months Old Boy, Familial Case

Conventional treatment was started during infancy and switched to Burosumab at 15.8 years of age due to profound worsening of biochemical and radiographic signs of rickets since onset of puberty at 13.5 years (starting dose 0.4mg/kg, 1 year follow-up: 0.6mg/kg). Serum phosphate and ALP normalized under low doses of Burosumab. Linear growth remained stable at -2SDS since the commencement of antibody treatment. Onset of frontal plane lower limb deformity was initially observed at 14 years of age. Under 12 months of Burosumab treatment, lower limb varus deformity and malrotation remained unaltered. Improvement of laboratory and rachitic physeal changes as well as improvement of knee pain was documented.

#### Case 6: 9 Years 11 Months Old Girl, Familial Case (Sister of Case 4), First Degree Relative with Confirmed PHEX Mutation

**Figure 8:**
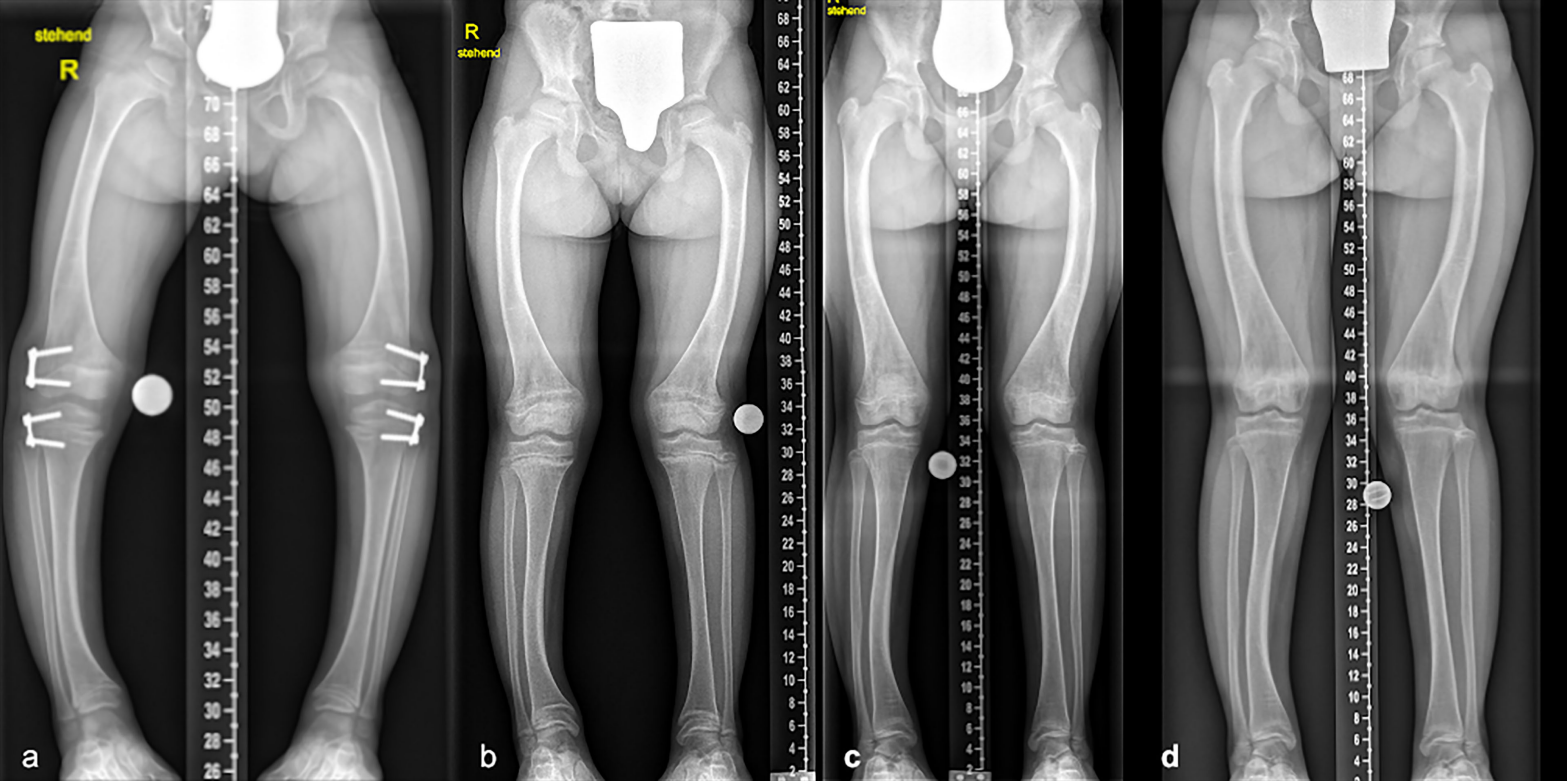
Radiographic progress of lower limb deformity in a 9 years 11 months old girl with familial XLH (case 6). Guided growth surgery was performed at age 5 years 1 month for varus deformity many years before Burosumab therapy **(A)**. Mechanical axis remained stable during follow-up before initiation of treatment **(B)**, at the one-year follow-up **(C)**, as well as the last follow-up 6.5 years after guided growth **(D)**.

**Figure 9:**
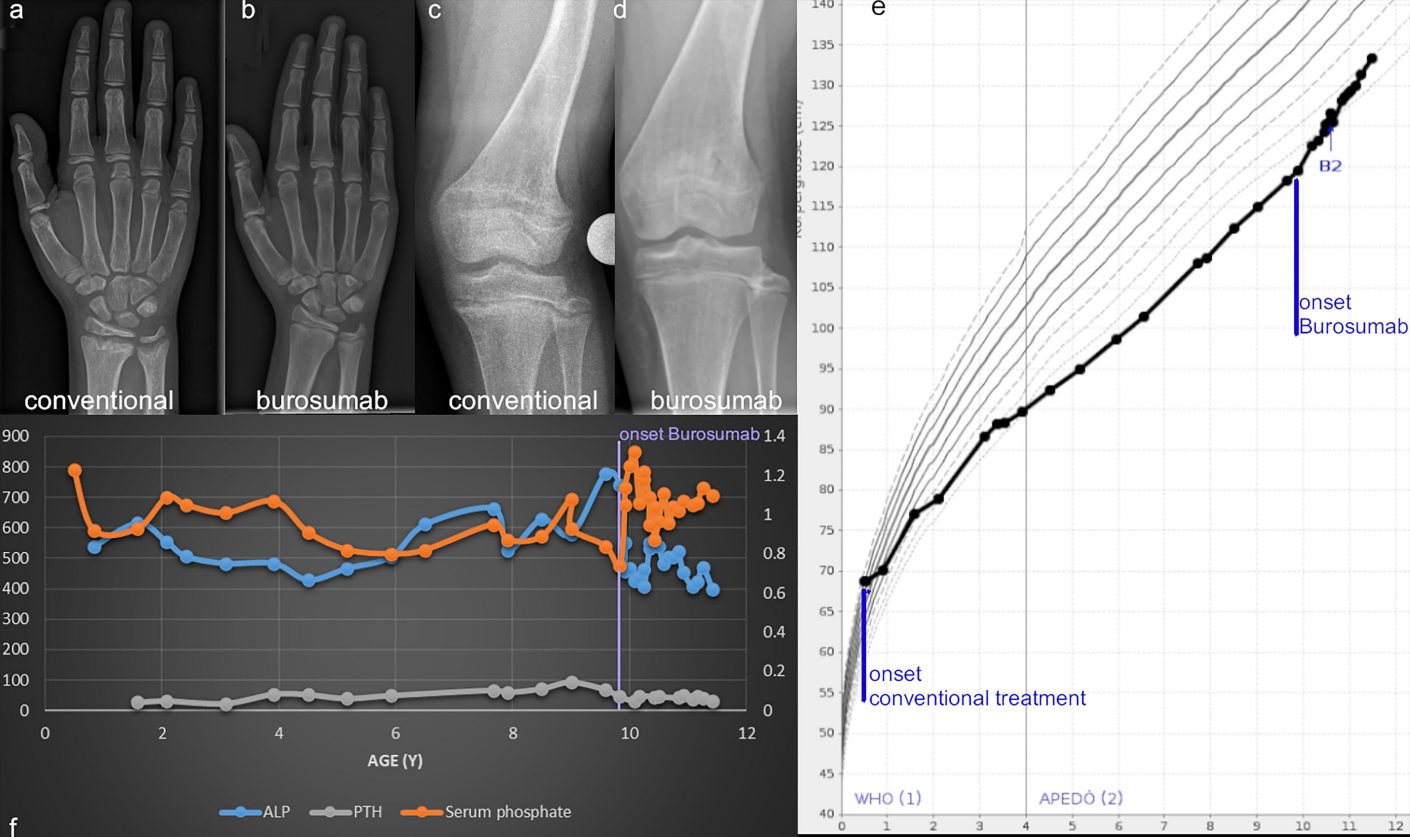
Wrist and knee radiographs obtained before therapy onset **(A, C)** and at the one-year follow-up show resolution of rickets **(B, D)** in case 6. Linear growth, onset of treatment and thelarche (B2) are marked on the Austrian reference growth chart for girls **(E)**. The course of relevant biochemical parameters shows increase of serum phosphate and normalization of ALP with onset of Burosumab treatment around 10 years of age **(F)**.

Conventional medical treatment with oral phosphate and alfacalcidol was initiated during infancy without major side effects. At age 5 years and 3 months a guided growth procedure on both knees on the distal lateral femur and proximal lateral tibia was performed due to varus malalignment of both legs (correction was achieved after 1 year 8 months - correction rate femur 0.45 and 0.7 degrees, tibia 0.55 and 0.6 degrees per month, MAD correction 1.9 and 2.45 mm per month) many years prior to initiation of Burosumab treatment (**Figure 8** and **Table 1**). Stable frontal lower limb despite residual femoral and tibial varus was observed in the long term follow up (5 years after guided growth procedure). However, clinically relevant maltorsion of both tibiae (gait deviation, frequent falling) was unaltered after 1 year of Burosumab despite excellent biochemical and radiographic response (**Figure 9**, starting dose 0.8mg/kg, 1 year follow-up: 1.8mg/kg).

#### Case 7: 8-Year-Old Girl, Familial Case

Conventional treatment with oral phosphates and calcitriol was commenced during early childhood. Due to gastrointestinal discomfort, Burosumab treatment was initiated at the age of 8 years and 2 months (starting dose 0.8mg/kg, 1 year follow-up: 1.4mg/kg). During conventional therapy, a decrease of tibial deformity from age 3 was observed, however, increase of severity of rachitic radiographic changes of the physis despite high patient compliance to conventional therapy led to initiation of Burosumab therapy. The patient underwent no prior surgeries. After one year of Burosumab, rachitic changes of physis receded completely, no frontal knee malalignment was observed and only mild maltorsion without complaints of gait disturbances was documented. Normalization of mild lateralization of mechanical axis right (although in this case normalization of mechanical axis and tibial deformity was observed in the years before Burosumab despite increase of rachitic changes). Gait analysis showed no improvement of intoeing gait during the study period.

## Discussion

Lower limb deformity and gait disturbances are major causes of life quality impairment in children and adults with XLH (3–7) but have been insufficiently identified as outcome parameters in prospective interventional studies so far. This study for the first-time reports standardized parameters of lower limb deformity and deformity correction rates of guided growth procedures in children with XLH undergoing Burosumab treatment. A comprehensive lower limb analysis showed no improvement of malrotation and frontal plane axis deviation (varus/valgus). Furthermore, this study for the first time describes new occurrences of lower limb deformity (knee valgus) and rebound of deformity despite Burosumab treatment.

Bone deformities are a typical sign of both dysplastic and metabolic skeletal disorders. In contrast to biochemical and radiographic assessment of rickets, the characterization of deformities requires standardized radiographic examination, torsional MRI, and gait analysis. Recently, specific deformities in XLH additionally to typical lower limb bowing have been shown by our group and others (4–6). These data revealed the limited use of planar radiographs as current standard of care to assess 3-dimensional deformities in XLH. Characteristically, XLH is associated with multi-apical lower limb deformities in different planes. Frontal plane deformities such as valgus or varus malalignment, often accompanied by sagittal diaphyseal bowing (procurvatum) have been described in this disorder recently (4, 5). Less information is available on disorder-specific sagittal plane deformities, typically procurvatum deformity of the femur, tibia, and fibula and on transverse plane deformities of the lower limb [maltorsion of femur and tibia/fibula (21)]. Although the need for lower limb deformity analysis in the evaluation of XLH patients was mentioned in several studies (4, 5, 9), detailed quantitative radiographic data on the influence of pharmacological treatment on lower limb development is lacking. Linglart et al. reported favorable results in treatment with Burosumab (9), the long leg standing radiographs were analyzed by a semiquantitative Global Impression Score (RGI-C) only, without any detailed deformity analysis. While RGI-C was validated in XLH to grade varus/valgus deformity (22), limitation to the frontal plane and unspecific classification into the levels mild, moderate, and severe without further specifications limits conclusions on the complex, 3-dimensional deformity phenotype in XLH.

Torsional MRI is a reliable method to analyze femoral and tibial maltorsion (16) and is widely used in centers for lower limb deformity correction. Tibial malrotation in XLH has been reported (1) but only recently quantified using gait analysis (4, 5). To the author’s knowledge, MRI data on femoral maltorsion in children with XLH has not been reported so far. While torsional MRI is not part of present XLH imaging protocols (23) it may gain importance considering the potentially high prevalence of severe maltorsion as suggested by our data.

The physiologic development of femoral and tibial torsion in children has been well characterized in the field of pediatric orthopedics: The femoral torsion is high after birth and continuously decreases until adolescence (17, 24). Tibial torsion is physiologically internally oriented at birth and progresses to external tibial torsion during growth (18). The data of our study group suggests that the developmental dynamics of tibial and femoral torsion during linear growth are substantially altered in XLH. Despite profound amelioration of radiologic and biochemical signs of rickets no evidence for reversibility of the torsional pathologies could be found in this case series after 12 months of Burosumab treatment. Furthermore, we observed the development of a unilateral valgus deformity despite 10 months of Burosumab treatment with satisfactory response. To our knowledge, this is the first case reported with onset of deformities under Burosumab treatment.

The finding of persistent deformities in this small, observational study raises questions regarding current hypotheses of hypophosphatemia as the main pathomechanism of growth plate affection and points to an additional intrinsic factor of bone development. A hypothesis of XLH as a skeletal disorder with dysplastic features and superimposed rickets would be supported by other non-rachitic affections of mineralized tissues in XLH with unclear pathomechanism: First, the specific dental phenotype with pathognomonic fistula and specific mineralization defects is not observed in nutritional rickets nor other types of hereditary hypophosphatemic rickets, pointing rather to a PHEX-specific effect than to a hypophosphatemia-associated etiology. A discrepancy between phosphate dependent- and independent effects has been demonstrated in Hyp mouse studies: In the murine model system, deficient mineralization could be corrected by inactivation of osteopontin (OPN) independently of correcting hypophosphatemia or FGF23 levels (25). Recently, the accumulation of OPN due to PHEX deficiency as a main pathomechanism and trigger for excessive FGF23 synthesis has been discussed (26). Thus, a dual hit hypothesis with local, PHEX/FGF23/OPN induced affection of mineralized tissues combined with systemic, FGF23-induced hypophosphatemia could be hypothesized. Among skeletal symptoms persistent to FGF23 blocking treatment, linear growth impairment has shown to be improved only to a very limited extent by Burosumab treatment despite resolution of rickets (8). While the precise cause of growth affection in XLH is unknown, data from Fgf23−/−/Hyp mice with normophosphatemia suggested a PHEX-specific effect on metaphyseal bone resembling a dyschondroplasic phenotype (27).

Also, in patients with achondroplasia, the most prevalent skeletal dysplasia, maltorsion has been described as a major component of lower limb deformity (28). In this study of achondroplasia patients, a decreased external tibial torsion similar to our XLH cohort in the present study was found. However, normal to increased femoral torsion and acetabular anteversion in achondroplasia (28) contrast our findings in XLH patients, revealing a markedly reduced femoral antetorsion. Other diagnostic groups such as cerebral palsy and patients with decreased musculoskeletal activity have been reported to develop an increased femoral neck anteversion (24).

The patients in this study group showed decreased external tibial torsion as well as severely decreased internal femoral torsion (MRI). As this is the first study to describe torsional deformity in children with gait analysis in combination with torsional MRI to date, no comparison between treatment effects on torsion development during growth can be performed. Furthermore, to the authors knowledge, there is no torsional MRI data available for nutritional rickets which would enable to draw distinction between dysplastic (skeletal dysplasia) and rachitic (hypophosphatemia) cause for lower limb deformity in patients with XLH.

Minimal invasive surgical treatment of planar lower limb deformities during linear growth has become standard of care in many pediatric orthopedic centers. Improvement of growth rate is crucial in pharmacological treatment in XLH, not only to increase body height, but also to improve the efficacy of guided growth procedures. While historically guided growth procedures in children with XLH receiving conservative treatment required prolonged treatment time until correction (case 6), three of our cases showed relatively fast correction during Burosumab treatment, resembling rates of correction reported in healthy children (19, 20). Although application of guided growth procedures has been reported in children with XLH (29), lack of data on correction rates hindered comparison to our data. Furthermore, recurrence of deformity after guided growth was reported (30), underlining the necessity of close follow-up until skeletal maturity. Additional data on correction rate, rebound risk and optimal age for surgery in children undergoing Burosumab therapy and guided growth procedures has yet to be collected.

In children with Burosumab therapy a faster correction rate was observed when compared to data in the literature [**Table 1** (19, 20)] but also compared to a very young child in this study receiving conventional treatment (case 6). However, the comparison of correction rate of guided growth is limited due to different severity of deformity and different age (19). Further studies are needed to evaluate an eventual synergistic effect of Burosumab and minimal invasive deformity correction, which potentially could reduce the numbers of osteotomies needed in the future. Regarding these invasive surgical procedures, bony healing after osteotomies in children with XLH was reported to be dependent on bone metabolism values (31). Case 1 did show adequate bony healing with Burosumab treatment, but no comparable data were reported in this age group. It might be speculated that the overall optimized phosphate homeostasis is beneficial for surgical outcomes and could lead to reduced numbers of interventions.

Spinal affection in patients with XLH has hardly been investigated. Conservative scoliosis therapy using braces, as applied in case 2, is usually recommended during linear growth as progression of scoliosis can occur with growth spurts (32). The clinical outcome might be positively influenced by Burosumab therapy in relation to advanced spinal growth. Increased awareness and inclusion of spine-specific parameters in future studies must be considered to understand and diagnose this rare but important symptom in children with XLH.

Some questions arose which require further studies for definite answers. Does commencement of Burosumab therapy at a younger age sufficiently prevent deformity development? Does Burosumab improve guided growth correction and decrease rate of deformity rebound and recurrence in children with XLH? To which amount is the skeletal affection in XLH caused primarily by dysplastic with superimposed rickets rather than a primarily rachitic disorder?

### Limitations

Although we report to date the most comprehensive analysis of lower limb deformity including radiographs, MRI and gait analysis in children receiving Burosumab therapy, several limitations must be considered. Our case series consisted of a small study group with a heterogeneous age range hindering any direct comparison among the included patients. Due to the lack of a control group, direct conclusions on treatment effects cannot be drawn. Despite standardized deformity analysis of anteroposterior radiographs, lack of lateral deformity analysis impeded evaluation of femoral or tibial sagittal plane bowing. In Austria, Burosumab therapy is restricted to patients with insufficient response or complications under conventional treatment. Thus, the study cohort may feature a more severe phenotype than other XLH cohorts. Treatment regimens of two externally monitored patients differed in terms of target biochemical markers, thus dosage was adapted differentially. Although correlation of gait analysis and torsional CT was reported, femoral values of rotation (gait analysis) and torsion (CT) correlate to a lesser extent than tibial values (33). However, additionally to the initial gait analysis, a torsional MRI was obtained in all children in this study. MRI was obtained at various time points within the study period and there was no follow up MRI at the one-year examination. To the author´s knowledge there are no pediatric reference values documented for femoral and tibial torsion based on MRI studies. Therefore, as mentioned in recent literature (24), historic values of radiographic and clinical examination were used for comparison (17, 18).

Unfortunately, no positive effects of Burosumab on lower limb deformity despite improved guided growth rates (3 cases) were observed in our prospective study. Further prospective studies with a preventive approach, a longer follow up, a larger cohort and an even more comprehensive deformity analysis are needed to evaluate the influence of Burosumab on the development of lower limb deformity.

## Conclusions

This case series for the first time reports detailed analyses of lower limb deformity and of guided growth procedures in children with XLH receiving Burosumab treatment. Improvements in biochemical and radiographic signs of rickets have not been accompanied with amelioration of lower limb deformities after 12 months of Burosumab treatment. First data on guided growth procedures raise a perspective of increased effectiveness under FGF23-blocking treatment. Future studies should include comprehensive deformity analysis in all three planes to address the essential aspect of lower limb deformity in XLH. A better comprehension of the dysplastic, non-rachitic features of XLH will help to improve persistent symptoms under Burosumab treatment and increase quality of life in this vulnerable cohort.

## Data Availability Statement

The raw data supporting the conclusions of this article will be made available by the authors for academic or scientific purposes, without undue reservation upon request to the corresponding author. Due to potential containing information that could compromise the privacy of research participants, a data sharing agreement has to be obtained in advance.

## Ethics Statement

The studies involving human participants were reviewed and approved by Ethikkommission der Wiener Krankenhäuser der Vinzenz Gruppe Gumpendorferstraße 108 1060 Wien. Written informed consent to participate in this study was provided by the participants’ legal guardian/next of kin. Written informed consent was obtained from the minor(s)’ legal guardian/next of kin for the publication of any potentially identifiable images or data included in this article.

## Author Contributions

GM, AS, AK, RG, CR, GH, and AR contributed to conception and design of the study. GM and AS wrote first draft of manuscript. AR wrote sections of the manuscript. GM, AS, GH, SP, and AR did clinical data collection. AK and AR performed statistical analysis. AK collected gait data. SP acquired MRI data and performed data analysis. GM, AK, AS, and AR performed overall data analysis. AR created graphical illustrations. All authors contributed to the article and approved the submitted version.

## Conflict of Interest

AR and GM received non-related honoraria from Kyowa Kirin for consultancy and scientific presentations. RG and CR received non-related honoraria from Nuvasive Inc. and Smith and Nephew for consultancy.

The authors declare that the research was conducted in the absence of any commercial or financial relationships that could be constructed as a potential conflict of interest.

## Publisher’s Note

All claims expressed in this article are solely those of the authors and do not necessarily represent those of their affiliated organizations, or those of the publisher, the editors and the reviewers. Any product that may be evaluated in this article, or claim that may be made by its manufacturer, is not guaranteed or endorsed by the publisher.

## Supplementary Material

Supplementary Table 1 Treatment-associated biochemical and radiographic parameters before and after 450 ± 146 days of Burosumab treatment. All values are displayed as mean ± SD.
